# Supplementary material for: Aircraft turnaround time dynamic prediction based on Time Transition Petri Net
Source: PLoS One. 2024 Jul 18;19(7):e0305237. doi: 10.1371/journal.pone.0305237 (PMC11257317; doi:10.1371/journal.pone.0305237)
Supplement: Attachment — Submitted filename: PONE-D-24-05331Comment.docx [file pone.0305237.s001.docx]

Manuscript Number: **PONE-D-24-05331**

Manuscript Title: **Aircraft Turnaround Time Dynamic Prediction Based on Time Transition Petri Net**

The study “**Aircraft Turnaround Time Dynamic Prediction Based on Time Transition Petri Net**” is the study proposes a new reliable research method for accurately predicting aircraft turnaround time. The objective of the study is very interesting, and the result is very useful to daily work of airports operation. However, its need some major/minor revision to improve. Some suggestion are as follow:

**Abstract:**

1. Abstract is reflected the manuscript. However order of some line should be revised.
2. The last line “This paper proposes …..turnaround time.” is similar explanation with second sentence “In this paper, by analyzing the aircraft… is purpose.” So, please revised as a one sentence. And the last line should be the useful of this study output or future research direction.
3. Line 24 to 25, Please avoided short form in abstract. Authors should write only the long form of RMS and MAE , E.g. “the root-mean-square error 3.75 minutes and the mean absolute error 3.40 minutes can be calculated.

**Introduction**

1. Figure 1, 2 and 6 should be revised to get clear resolution like other figures.
2. The study objective is explained and clearly, but the significance of this work and structure of manuscript should be write in last part of this section.

**Material and Method**

1. Authors should mention about data information in details before the explanation of methods. Not is result section.
2. What historical data is used for the study? By time or total number of events?
3. Authors should consider other impact on airport operation management in their calculation such as weather delay situation in future study.

**Result and discussion**

1. Figure 7 should be revised for clear resolution.
2. Please move two square error equations to method section. Result section should be only results of everything.

**General Comment**

1. Authors should mention the airports list or location map where the data are taken to get more reader interesting.
2. Please put the section number to identify the major section and revised some section titles, For E.g. “Evaluate the prediction level of each link” is like a sentence or figure caption, not a title. It should be only ‘Evaluate the prediction level”.
3. The study objective is proposed to new methodology for future operation study, thus please highlight the method procedure in manuscript. Please move minor result table to supplementary section. And if possible revised these tables to figures to put in manuscript.
