# Supplementary material for: Aircraft turnaround time dynamic prediction based on Time Transition Petri Net
Source: PLoS One. 2024 Jul 18;19(7):e0305237. doi: 10.1371/journal.pone.0305237 (PMC11257317; doi:10.1371/journal.pone.0305237)
Supplement: Attachment — Submitted filename: Response to Reviewers-20240422.docx [file pone.0305237.s002.docx]

Manuscript Number: **PONE-D-24-05331**

Manuscript Title: **Aircraft Turnaround Time Dynamic Prediction Based on Time Transition Petri Net**

Dear Editor and Reviewer:

I am very honored to receive your reply. Thank you very much for your valuable comments on the paper. The major comment as follows:

1． **Please ensure that your manuscript meets PLOS ONE's style requirements, including those for file naming. The PLOS ONE style templates can be found at**

**https://journals.plos.org/plosone/s/file?id=wjVg/PLOSOne_formatting_sample_main_body.pdf and**

**https://journals.plos.org/plosone/s/file?id=ba62/PLOSOne_formatting_sample_title_authors_affiliations.pdf**

Thanks to the editor's reminding, I strictly checked the style requirements of the two documents again and renamed the documents.

2． **Please note that PLOS ONE has specific guidelines on code sharing for submissions in which author-generated code underpins the findings in the manuscript. In these cases, all author-generated code must be made available without restrictions upon publication of the work. Please review our guidelines at**

**https://journals.plos.org/plosone/s/materials-and-software-sharing#loc-sharing-code and ensure that your code is shared in a way that follows best practice and facilitates reproducibility and reuse.**

The core code will be provided below, but the data has confidentiality requirements before the project is made public, so it is not convenient to provide the source data.

```
import numpy as np
import pandas as pd
import matplotlib.pyplot as plt
from sklearn.metrics import mean_squared_error
from sklearn.linear_model import BayesianRidge
from sklearn.preprocessing import StandardScaler
from sklearn.model_selection import cross_val_score, train_test_split

def open_file_data():
    f = open('***.csv', encoding='UTF-8')
    df = pd.read_csv(f, index_col=0)
    # print(df1)
    X = list()
    y = list()
    for i in df.index - 1:
        X.append(df.iloc[i, 0:-1].to_numpy())
        y.append(df.iloc[i, -1:].to_numpy())
    # print(y)
    X = np.array(X)
    y = np.array(y)
    return X, y
def algorithm_br():
    """ ------------------------ BayesianRidge ------------------------
    def __init__(self, n_iter=300, tol=1.e-3, alpha_1=1.e-6, alpha_2=1.e-6,
                 lambda_1=1.e-6, lambda_2=1.e-6, compute_score=False,
                 fit_intercept=True, normalize=False, copy_X=True,
                 verbose=False):
     --------------------------------------------------------------------------- """
    print('BayesianRidge')
    global br
    br = BayesianRidge(compute_score=True)
    br.fit(X_train, y_train)

    """ -------------------- R^2 --------------------- """
    br_test_R2 = str(round(br.score(X_test, y_test), 3))
    print('BayesianRidge Test R2 = %s' % br_test_R2)
    """ ------------------ RMSE -------------------- """
    br_test_RMSE = mean_squared_error(y_true=y_test, y_pred=br.predict(X_test), squared=False)
    print('BayesianRidge Test RMSE = %.3f' % br_test_RMSE)
def mat_plt_br():
    plt.figure(figsize=(5, 5))
    plt.rcParams['font.sans-serif'] = ['SimSun']
    plt.rcParams['axes.unicode_minus'] = False
    plt.scatter(y_train, br.predict(X_train), alpha=0.5, color='blue', label='training')
    plt.scatter(y_test, br.predict(X_test), alpha=0.5, color='red', label='test')
    plt.legend(fontsize=14)
    plt.xticks(fontsize=14)
    plt.yticks(fontsize=14)
    plt.xlabel('', fontsize=14)
    plt.ylabel('', fontsize=14)
    plt.show()

if __name__ == "__main__":
    X, y = open_file_data()
    # X = StandardScaler().fit_transform(X)
    X_train, X_test, y_train, y_test = train_test_split(X, y, test_size=0.10, random_state=1)
    print(X_train)
    print(X.shape)
    algorithm_br()
    mat_plt_br()
    My_Predict_Data = np.array([[*,*,*,*,*,*,*,*,*,*,*,*]])
    print("Predict: " + str(br.predict(My_Predict_Data)))
```

3． **We note that the grant information you provided in the ‘Funding Information’ and ‘Financial Disclosure’ sections do not match. When you resubmit, please ensure that you provide the correct grant numbers for the awards you received for your study in the ‘Funding Information’ section.**

Thank you for your reminding, it has been resubmitted.

4． **Thank you for stating the following financial disclosure:" the National Key Research and Development Project (2021YFB1600500-2021YFB1600502)."**

**Please state what role the funders took in the study. If the funders had no role, please state: ""The funders had no role in study design, data collection and analysis, decision to publish, or preparation of the manuscript. ""If this statement is not correct you must amend it as needed. Please include this amended Role of Funder statement in your cover letter; we will change the online submission form on your behalf.**

The funders had no role in study design, data collection and analysis, decision to publish, or preparation of the manuscript.

The cover letter has been revised.

5． **We note that your Data Availability Statement is currently as follows: All relevant data are within the manuscript and its Supporting Information files.**

**Please confirm at this time whether or not your submission contains all raw data required to replicate the results of your study. Authors must share the “minimal data set” for their submission. PLOS defines the minimal data set to consist of the data required to replicate all study findings reported in the article, as well as related metadata and methods (**[**https://journals.plos.org/plosone/s/data-availability#loc-minimal-data-set-definition**](https://journals.plos.org/plosone/s/data-availability#loc-minimal-data-set-definition)**).**

I am very sorry to reply you that the data in this article is owned by a third party, and there are many support units involved in the aircraft's turnaround, and the support methods and specific support time and other information of each unit are kept confidential.

6． **PLOS requires an ORCID iD for the corresponding author in Editorial Manager on papers submitted after December 6th, 2016. Please ensure that you have an ORCID iD and that it is validated in Editorial Manager. To do this, go to ‘Update my Information’ (in the upper left-hand corner of the main menu), and click on the Fetch/Validate link next to the ORCID field. This will take you to the ORCID site and allow you to create a new iD or authenticate a pre-existing iD in Editorial Manager. Please see the following video for instructions on linking an ORCID iD to your Editorial Manager account:** [**https://www.youtube.com/watch?v=_xcclfuvtxQ**](https://www.youtube.com/watch?v=_xcclfuvtxQ)

Thank you for reminding me that ORCID iD has been added.

**Reviewer #1:**

**Abstract:**

1. **Abstract is reflected the manuscript. However, order of some line should be revised.**

The order of description of abstracts has been changed based on article order. Move the " According to the actual landing time of the flight, the aircraft turnaround time is predicted" to the end of the "…Bayesian theorem is designed", as you can see in line 19 of the article. Then, the dynamic prediction steps are described again.

2. **The last line “This paper proposes …turnaround time.” is similar explanation with second sentence “In this paper, by analyzing the aircraft… is purpose.” So, please revised as a one sentence. And the last line should be the useful of this study output or future research direction.**

The two sentences have been merged and the usefulness of the research findings has been added to the last line. "This paper contributes to the improvement of flight punctuality rate and airport clearance level" this sentence is added to the end.

3. **Line 24 to 25, Please avoided short form in abstract. Authors should write only the long form of RMS and MAE , E.g. “the root-mean-square error 3.75 minutes and the mean absolute error 3.40 minutes can be calculated.**

Thank you for reminding me that it has changed.

**Introduction:**

1. **Figure 1, 2 and 6 should be revised to get clear resolution like other figures.**

The changed image is as follows:

**Fig 1. Flight turnaround operation process**

**Fig 2. Petri network concurrency relationship description**

**Fig 6. Dynamic prediction steps of turnaround time**

2. **The study objective is explained and clearly, but the significance of this work and structure of manuscript should be write in last part of this section.**

The last paragraph of the introduction has been modified as follows:

Under the background of increasingly busy airports, it is of great significance to provide passengers with reliable travel schedules and improve the service performance of airports and airlines. This paper first sorted out the necessary support activities of the flight turnaround through the station, thus establishing the Time Transition Petri Net (TTPN), and then combined Bayesian theorem to predict the flight turnaround time, analyzed and verified the prediction model with actual data, and finally proved that the prediction method has certain reference value.

**Material and Method:**

1. **Authors should mention about data information in details before the explanation of methods. Not is result section.**

Explanations have been added to the data sources：

I am very sorry to reply you that the data in this article is owned by a third party, and there are many support units involved in the aircraft's turnaround, and the support methods and specific support time and other information of each unit are kept confidential.

2. **What historical data is used for the study? By time or total number of events?**

The data source of this article is the operational data of a hub airport in China. Effective operational data for 2023 was collected, and data cleaning and analysis were conducted on this basis.

3. **Authors should consider other impact on airport operation management in their calculation such as weather delay situation in future study.**

Personally, I quite agree with you. At present, many scholars' articles have confirmed that climate has a great impact on flight delay. But how to extend the working time of various security departments still needs to be studied, which is also the direction of my future efforts.

**Result and discussion:**

1. **Figure 7 should be revised for clear resolution.**

**Fig 7. Data training situation**

2. **Please move two square error equations to method section. Result section should be only results of everything.**

Move the two square error equations to the method section and add them to step 6 as the last step in the prediction step.

Step 6: According to the results of the test set and training set, the prediction level is evaluated by calculating the root-mean-square error and the mean absolute error.

|  | $RMSE\left(S,\mathop{S}\limits^{\wedge} \right)=\sqrt{\frac{1}{n}\sum_{t=1}^{n} \left(s_{t}-\mathop{s_{t}}\limits^{\wedge} \right)^{2}}$ | （5） |
| --- | --- | --- |
|  | $MAE\left(S,\mathop{S}\limits^{\wedge} \right)=\frac{1}{n}\sum_{t=1}^{n} \left(s_{t}-\mathop{s_{t}}\limits^{\wedge} \right)$ | （6） |

**General Comment**

1. **Authors should mention the airports list or location map where the data are taken to get more reader interesting.**

Thank you for your reminding. Data cannot be shared publicly because of they are owned by a third party, and there are many support units involved in the aircraft's turnaround, and the support methods and specific support time and other information or each unit are kept confidential.

2. **Please put the section number to identify the major section and revised some section titles, For E.g. “Evaluate the prediction level of each link” is like a sentence or figure caption, not a title. It should be only ‘****Evaluate the prediction level”.**

1.Introduction

2.The turnaround process description

2.1 Time Transition Petri Net (TTPN)

2.2 The turnaround process modeling

3.Dynamic prediction of turnaround time

3.1 Study on the Parameters

3.2 Dynamic prediction steps of turnaround time

4.Results

4.1 Data processing

4.2 Flight verification analysis

4.3 Evaluate the prediction level

5.Conclusions

3. **The study objective is proposed to new methodology for future operation study, thus please highlight the method procedure in manuscript. Please move minor result table to supplementary section. And if possible, revised these tables to figures to put in manuscript.**

Step 1: According to the operation service links represented by each transition in TTPN, the prior probability distribution normal fitting of each link is carried out by using historical data, and the joint distribution law between the related links is obtained.

Step 2: The Shapiro-Wilke test is used to confirm whether the prior probability normal fitting is valid, and the conditional probability distribution function is determined.

Step 3: Take the maximum probability of each link as the initial value, combined with Monte Carlo simulation to determine the range of conditional probability for each round ;

Step 4: According to the logical relationship of the operation service link in TTPN, the Monte Carlo simulation value of each round is used as the duration of the subsequent link change, and the order of the link is updated.

Step 5: With the continuous progress of each process, repeat the prediction of the completion time of the follow-up link. If the simulation order is less than 12, go to step 3.

Step 6: According to the results of the test set and training set, the prediction level is evaluated by calculating the root-mean-square error and the mean absolute error.

|  | $RMSE\left(S,\mathop{S}\limits^{\wedge} \right)=\sqrt{\frac{1}{n}\sum_{t=1}^{n} \left(s_{t}-\mathop{s_{t}}\limits^{\wedge} \right)^{2}}$ |  |
| --- | --- | --- |
|  | $MAE\left(S,\mathop{S}\limits^{\wedge} \right)=\frac{1}{n}\sum_{t=1}^{n} \left(s_{t}-\mathop{s_{t}}\limits^{\wedge} \right)$ |  |

**Reviewer #2:**

1. **Add more applications related to proposed mathematical modeling.**

Thank you for your reminding, the relevant content has been added to the introduction:

Petri Nets are used in the analysis of operation processes in various industries, such as logistics warehousing, manufacturing and assembly, signal communication. It has a good ability to describe discrete events.

2. **Add more physical explanation about the mathematical modeling.**

The following two changes have been made：

1) The TTPN can be defined as $\left(t_{1},t\to\right)$, $t_{1}$is the initial time. The TTPN contains the departure conditional probability distribution at the initial time $P\left(t_{i+1}\left|t_{i}\right.\right)$. Formula 2 shows that when $t_{i}$ event occurs, the probability of causing $t_{i\text{+}1}$ event to occur is calculated to find out what is most likely to cause it to occur.

|  | $P\left(t_{i+1}\left\vert t_{i}\right.\right)=\frac{P\left(t_{i+1}\right)P\left(t_{i}\left\vert t_{i+1}\right.\right)}{\sum_{j=i+1}^{n} P\left(t_{j}\right)P\left(t_{i}\left\vert t_{j}\right.\right)}$ | （2） |
| --- | --- | --- |

Where, $\left(t_{1},t\to\right)$: the evolution mechanism of departure process. $P\left(t\to\left|t_{1}\right.\right)$: The distribution between variables after the process has been updated.

According to the TTPN structure of the aircraft turnaround process, the conditional probability density of the turnaround time prediction time can be obtained according to the chain rule.

|  | $\text{f}\left(\text{t}_{1}\text{,t}_{2}\text{,...t}_{12}\right)\text{=f}\left(\text{t}_{12}\left\vert\text{t}_{11}\text{,...t}_{1}\right.\right)\text{...f}\left(\text{t}_{2}\left\vert\text{t}_{1}\right.\right)\text{f}\left(\text{t}_{1}\right)$ | （3） |
| --- | --- | --- |

2) Where, $x_{\left(i\right)}$: The $i$th minimum number in the sample.$\mathop{X}\limits^{\_\_}$: Average number of sample data. Where the numerator, when it is even, it is. When it is odd, it is.

3. **The paper should be prepared according to the instructions of this journal.**

Thank you for your reminding. I have modified the article format, line spacing and size according to the requirements of this journal.

4. **The title of the manuscript should be more concise.**

1.Introduction

2.The turnaround process description

2.1 Time Transition Petri Net (TTPN)

2.2 The turnaround process modeling

3.Dynamic prediction of turnaround time

3.1 Study on the Parameters

3.2 Dynamic prediction steps of turnaround time

4.Results

4.1 Data processing

4.2 Flight verification analysis

4.3 Evaluate the prediction level

5.Conclusions

5. **Author should justify what are the novel contributions of their study using "DTM" approach.**

I'm sorry I'm not familiar with DTM method. Do not understand how to apply the DTM method. In the future research, I will use more mathematical methods to further analyze the simulation results.

6. **The following studies would be useful for the readers:**

**a) https://doi.org/10.1007/s11771-023-5398-1**

**b) https://doi.org/10.1080/17455030.2022.2123115**

**c) https://doi.org/10.1142/S0217984921505527**

**d)** [**https://doi.org/10.3390/math9172139**](https://doi.org/10.3390/math9172139)

Thank you very much. I have benefited a lot from these papers.
